# Evaluating the Ability of Open-Source Artificial Intelligence to Predict Accepting-Journal Impact Factor and Eigenfactor Score Using Academic Article Abstracts: Cross-sectional Machine Learning Analysis

**DOI:** 10.2196/42789

**Published:** 2023-03-07

**Authors:** Carmelo Macri, Stephen Bacchi, Sheng Chieh Teoh, Wan Yin Lim, Lydia Lam, Sandy Patel, Mark Slee, Robert Casson, WengOnn Chan

**Affiliations:** 1 Discipline of Ophthalmology and Visual Sciences The University of Adelaide Adelaide Australia; 2 Department of Ophthalmology The Royal Adelaide Hospital Adelaide Australia; 3 Department of Radiology The Royal Adelaide Hospital Adelaide Australia; 4 College of Medicine and Public Health Flinders University Adelaide Australia

**Keywords:** journal impact factor, artificial intelligence, ophthalmology, radiology, neurology, eye, neuroscience, impact factor, research quality, journal recommender, publish, open source, predict, machine learning, academic journal, scientometric, scholarly literature

## Abstract

**Background:**

Strategies to improve the selection of appropriate target journals may reduce delays in disseminating research results. Machine learning is increasingly used in content-based recommender algorithms to guide journal submissions for academic articles.

**Objective:**

We sought to evaluate the performance of open-source artificial intelligence to predict the impact factor or Eigenfactor score tertile using academic article abstracts.

**Methods:**

PubMed-indexed articles published between 2016 and 2021 were identified with the Medical Subject Headings (MeSH) terms “ophthalmology,” “radiology,” and “neurology.” Journals, titles, abstracts, author lists, and MeSH terms were collected. Journal impact factor and Eigenfactor scores were sourced from the 2020 Clarivate Journal Citation Report. The journals included in the study were allocated percentile ranks based on impact factor and Eigenfactor scores, compared with other journals that released publications in the same year. All abstracts were preprocessed, which included the removal of the abstract structure, and combined with titles, authors, and MeSH terms as a single input. The input data underwent preprocessing with the inbuilt ktrain Bidirectional Encoder Representations from Transformers (BERT) preprocessing library before analysis with BERT. Before use for logistic regression and XGBoost models, the input data underwent punctuation removal, negation detection, stemming, and conversion into a term frequency-inverse document frequency array. Following this preprocessing, data were randomly split into training and testing data sets with a 3:1 train:test ratio. Models were developed to predict whether a given article would be published in a first, second, or third tertile journal (0-33rd centile, 34th-66th centile, or 67th-100th centile), as ranked either by impact factor or Eigenfactor score. BERT, XGBoost, and logistic regression models were developed on the training data set before evaluation on the hold-out test data set. The primary outcome was overall classification accuracy for the best-performing model in the prediction of accepting journal impact factor tertile.

**Results:**

There were 10,813 articles from 382 unique journals. The median impact factor and Eigenfactor score were 2.117 (IQR 1.102-2.622) and 0.00247 (IQR 0.00105-0.03), respectively. The BERT model achieved the highest impact factor tertile classification accuracy of 75.0%, followed by an accuracy of 71.6% for XGBoost and 65.4% for logistic regression. Similarly, BERT achieved the highest Eigenfactor score tertile classification accuracy of 73.6%, followed by an accuracy of 71.8% for XGBoost and 65.3% for logistic regression.

**Conclusions:**

Open-source artificial intelligence can predict the impact factor and Eigenfactor score of accepting peer-reviewed journals. Further studies are required to examine the effect on publication success and the time-to-publication of such recommender systems.

## Introduction

Peer review processes for scientific articles can be time-consuming. When target journals are not identified appropriately, the process may result in multiple resubmissions and delays in disseminating research findings [1]. In addition, given more than half of submitted conference abstracts are not subsequently published [2] and the most common reason for not publishing abstract results as full publication is lack of time [3], there is a need to increase the efficiency of the publication process for researchers. Recommender systems may be useful in increasing full publication rates by guiding researchers to journals with a higher likelihood of acceptance. The demand for journal recommender systems is highlighted by major publishers’ proliferation of these services [4-9]. However, these publisher-specific services recommend only within a publisher’s library of journals and use proprietary methods. Open-source solutions may provide alternative means of journal suggestions that are cross-platform. However, previous applications have been limited to computer science and biomedical domains.

Different approaches to the problem of journal recommendation have included content-based filtering recommendation, collaborative filtering–based recommendation, network-based filtering, and hybrid combinations of these [10]. Content-based filtering recommendation is the earliest consideration for most researchers in deciding where to submit an article. Content-based filtering uses the content of an article abstract to suggest journal recommendations. Early algorithms used non–deep learning methods to suggest journals using article abstracts. For example, eTBLAST extracts a weighted keyword set from the input article abstract to gather the top 400 most similar articles in MEDLINE [11]. A novel sentence alignment algorithm refines the rank order of similar records and computes a z score aggregated per journal [11]. Another example is Jane (journal or author name estimator), which similarly uses a vector space approach to identify similar articles based on an article abstract and uses a k-nearest neighbor approach to determine the author list [12]. Jane’s approach performed well and showed consistent improvement over eTBLAST [12]. A range of newer algorithms, including those incorporating deep learning techniques, have shown increased accuracy. Wang et al’s [13] “publication recommender system” uses term frequency-inverse document frequency (TD-IDF), chi-squared feature selection, and softmax regression classification to suggest journals for computer science publications. This algorithm was subsequently extended by Huynh et al [14] using multilayer perceptrons as a classifier and by Nguyen et al [15] introducing a one-dimensional convolutional neural network. Accuracy measured on the same computer science data set showed increased improvement with the incorporation of artificial intelligence methods. Further artificial intelligence methods with increasing accuracy have also been developed [16,17], demonstrating the potential for artificial intelligence algorithms to improve journal recommendation systems. However, the algorithms have been limited to the computer, science, mathematics, and biomedicine domains, and there is a lack of applications for clinical journals.

There are additional factors, such as journal reputation, to consider when selecting appropriate target journals for scientific articles [18-20]. Two common indicators of journal reputation are the impact factor and Eigenfactor scores [21,22]. These metrics provide a quantitative indicator of journal reputation. Both scores are based on the number of citations received by articles published in the given journal [23]. Current algorithms have not incorporated these metrics into their recommendation algorithms.

This pilot study aimed to assess the performance of artificial intelligence when applied to article abstracts in the prediction of accepting journal impact factor and Eigenfactor score tertile.

## Methods

### Data Collection

PubMed-indexed articles published between 2016 and 2021 were identified with the Medical Subject Heading (MeSH) terms “ophthalmology,” “radiology,” and “neurology.” These fields were selected to evaluate journal recommendations in a clinical domain compared to computer science or biomedicine. These 3 fields are often related and provide a broader scope compared to a single discipline. Titles, abstracts, author lists, and MeSH terms were collected. The journal’s ISSN for each article was identified, along with the associated journal impact factor. Articles without a specified journal ISSN or articles that could not be linked to an impact factor were excluded from the study. Journal impact factor and Eigenfactor scores were listed by the 2020 Clarivate Journal Citation Report. The journals included in the study were allocated percentile ranks based on the impact factor and Eigenfactor scores, compared with other journals that released publications in the same year. The impact factor is calculated by dividing the current year citations by the source items published in that journal during the previous 2 years. The Eigenfactor score is a weighted measure of the number of times articles from the journal published in the past 5 years has been cited in the Journal Citation Report year, eliminating self-citations. The calculation algorithm is freely available [24]. These complementary measures were used, as the impact factor reflects popularity as opposed to the Eigenfactor score that reflects prestige or trustworthiness [25].

### Text Preprocessing

All abstracts underwent a process involving the removal of abstract structure (eg, line breaks) and headings (eg, “background” and “objective”). Titles, abstracts, authors, and MeSH terms were combined and used as a single input. The input data underwent preprocessing with the inbuilt ktrain Bidirectional Encoder Representations from Transformers (BERT) preprocessing library before analysis with BERT [26]. Ktrain is a wrapper of the TensorFlow Keras library, which was used to conduct these BERT analyses. With this inbuilt BERT preprocessing model, a maximum length of 400 words was specified, which was selected due to the length of included texts and the processing requirements of the analysis.

Before use for logistic regression and XGBoost models, the input data underwent punctuation removal, negation detection, as well as stemming and conversion into a term frequency-inverse document frequency (TF-IDF) array. Punctuation removal involved the removal of all nonletter and nonnumerical characters with regular expressions. Negation detection was employed using the 19 libraries [27]. This negation detection uses a prespecified set of negating terms, following which subsequent terms will be flagged as negated. Word stemming was conducted with the Natural Language Toolkit Porter stemmer function. The Porter stemming approach involves the removal of common word suffixes and inflections. Finally, the text was converted into TF-IDF arrays using scikit-learn [28]. The n-gram range was allowed to vary from n-grams of 1 segment in length to 3 segments during training. Ultimately, the used n-gram range was 1 to 3. A similar approach was employed for the maximum number of features, which was set at 10,000.

Following this preprocessing, data were split into training and testing data sets randomly. This separation was conducted with a 3:1 train:test ratio. This train-test split was performed once. These preprocessing methods and the models used below were selected, as similar methods had previously proved effective in analyzing scientific abstracts [29].

### Model Development and Classification Experiments

Models were developed to predict whether a given article would be published in a first, second, or third tertile journal (0-33rd centile, 34th-66th centile, or 67th-100th centile), as ranked either by impact factor or Eigenfactor score. BERT, XGBoost, and logistic regression models were developed on the training data set before evaluation on the hold-out test data set. The logistic regression model was selected to provide a baseline for classification accuracy. This model was trained on the training data set and then evaluated on the test data set using the default values for all hyperparameters as in the scikit-learn library. The XGBoost and BERT algorithms were developed on the training set using 5-fold cross-validation. During this process, multiple XGBoost hyperparameters were varied, including the number of estimators, maximum depth, and learning rate. Following experimentation, there were no significant improvements in model performance on the training data set; therefore, library defaults were also used for this model. The BERT model was developed using ktrain. During the development of the BERT model, learning rate, epochs and batch size were allowed to vary. The hyperparameters used were a batch size of 3, a learning rate of 2×10^–5^, and 3 epochs. Following training, these models were evaluated on a hold-out test data set (to minimize the risk of data leakage).

The primary outcome was overall classification accuracy for the best-performing model in the prediction of accepting journal impact factor tertile. In addition, 2 random examples were selected to demonstrate instances of correct and incorrect classification to improve interpretability. Open-source Python libraries were used for analysis, including XGBoost, scikit-learn, TensorFlow, Natural Language Toolkit, and ktrain [26-28,30,31].

## Results

### Journal and Article Characteristics

There were 10,813 articles included in the study. These articles were from 382 unique journals. The median impact factor for the journals was 2.117 (IQR 1.102-2.622). The median Eigenfactor score was 0.00247 (IQR 0.00105-0.03).

### Impact Factor Prediction

The BERT model achieved the highest classification accuracy of 75.0%. XGBoost returned a classification accuracy of 71.6%. Logistic regression, which served as a baseline classification accuracy, achieved a classification accuracy of 65.4%.

### Eigenfactor Prediction

Performance in Eigenfactor prediction was similar to impact factor prediction. The BERT model achieved a classification accuracy of 73.6%. XGBoost and logistic regression returned 71.8% and 65.3% classification accuracies, respectively. In this instance, logistic regression again provided a baseline classification accuracy.

### Interpretability

**Figure 1:**
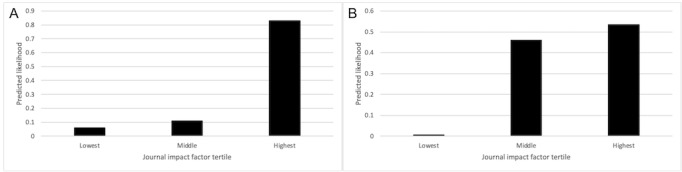
(A) Example of predicted impact factor tertile for correct classification. (B) Example of predicted impact factor tertile for incorrect classification.

An article by Kubak et al [32] was selected as a random example of a correct classification. When provided with the title, abstract, authors, and MeSH terms, as may have been provided at the time of submission, the BERT model correctly predicted that the article would be published in a highest tertile journal (*Annals of Translational Medicine*, impact factor of 3.932; Figure 1A). Conversely, the article by Gao et al [33] was predicted to be published in a highest tertile journal and was published in a middle tertile journal (*Journal of Ophthalmology*, impact factor 1.909; Figure 1B) [33].

## Discussion

### Principal Results

This study demonstrates the ability of machine learning classifiers to predict the impact factor and Eigenfactor score of journals that will accept a given peer-reviewed article based on an abstract in the often-related fields of ophthalmology, neurology, and radiology. The BERT model was most effective in this study, achieving an impact factor and Eigenfactor score tertile classification accuracy of 75.0% and 73.6%, respectively. Artificial intelligence may assist researchers in selecting journals with a higher probability of acceptance, with the potential for increasing rates of full publication or reducing time to publication. Further studies are required to assess this.

### Limitations

The algorithms involved in this project have similar limitations to existing recommender systems. Since the models have learned from previous publications, they may perpetuate existing research trends regarding which studies are or are not considered a high priority. Similarly, the models may not have been exposed to new terminology associated with novel research, with resultant reduced prediction accuracy until the model is trained with these terms.

Ongoing research in this area may develop similar models that encompass additional fields. Research examining the use of such models to ascertain whether their use increases submission efficiency is required. In addition, future studies examining the development and use of other scientific writing recommender systems may be beneficial.

### Comparison With Prior Work

Content-based journal recommender systems have evolved with increasing accuracy with the introduction of artificial intelligence and deep learning algorithms. Compared to the major prior algorithms that recommend specific journals, our approach recommends a journal impact factor tertile. This may be beneficial in smaller domains, such as a clinical speciality, where the journals and their hierarchy are well known to researchers. Deciding which impact factor or Eiegenfactor score tertile to target may be more informative than a single journal. Other available major prior algorithms vary in their approaches. As mentioned, the Publication Recommender System uses TF-IDF and chi-squared feature selection and logistic regression as a classifier [13]. The accuracy of the classifier model for correctly recommending a journal within the top 3 suggestions was 0.6137. This algorithm was extended by Huynh et al [14] in their Scientific Submission Recommendation System for Computer Science (S2RSCS) algorithm using multilayer perceptrons as classifiers. Evaluated on the Wang et al’s [13] computer science data set, they achieved an accuracy of 0.8907 when using the title, abstract, and keywords as input to predict the top 3 computer science journals. Later, Nguyen et al [15] introduced S2CFT, a variation on this technique using deep learning that demonstrated improved accuracy. FastText embeddings were combined with a 1-dimensional convolutional neural network to produce a matching score model. The output vector from this model was averaged with the S2RSCS model output vector to create a new vector to calculate a matching score. The top-3 accuracy achieved on the Wang et al [13] computer science data set using title, abstract, and keywords was 0.9021 [15]. Deep learning is also used in Feng et al [34]’s Pubmender algorithm, which uses pretrained word2vec to construct the initial feature space, with a subsequent deep convolutional neural network to achieve a high-level representation of article abstracts [34]. A softmax regression model was used to recommend the top 3 biomedical journals. The accuracy of the Pubmender model for the top 3 biomedical journals was 0.71. Nguyen et al’s [16] “paper submission recommendation using mixtures of transformer encoders” (PSRMTE) algorithm is another framework that has shown further increased performance. The PSRMTE method uses different bidirectional transformer encoders and combines them using the mixtures of transformer encoders (MTE) technique to train a classification model to estimate the conditional probability of a given paper submission belonging to a journal. Matching scores between the paper submitted and each journal are used to return the top journals. PSRMTE performance was evaluated using combinations of title, abstract, and the list of keywords. The top-3 accuracy of the MTE algorithm on the Wang et al [13] computer science data set using the abstract and keywords was 0.9225. The excellent results of the MTE algorithm demonstrate the power of transformer encoders in this task. The extensive benefits of transformer encoders [16] and their demonstrated effectiveness explain why the BERT model performed best among our specified algorithms. Another algorithm, Content and Bipartite Graph to Recommend Journals for Submission (CBGJRS), is a hybrid algorithm that also uses transformer encoders [17]. CBGJRS uses a “BERT pre-training model to obtain a vector representation of articles by feature learning from abstracts at the text level and a self-coding network to obtain a vector representation of journals, followed by a scoring function and a softmax classifier to achieve journal recommendations.” [17] The accuracy of the CBGJRS approach to recommend a set of top 3 journals was 0.9536 using a closed data set and 0.7206 on a larger validation data set. The authors conclude that adding journal features in their hybrid approach improves performance. Our results suggest that inputs such as citation metrics could be incorporated into future algorithms. Further work on journal recommendations for clinical journals is required to show the cross-domain potential of these algorithms. This would require standardized data sets for clinical journals to allow for algorithm comparison, as has been used in evaluating algorithms in other domains.

### Conclusions

In conclusion, we demonstrated the pilot ability of open-source artificial intelligence classifiers to predict the accepting-journal impact factor and Eigenfactor score of an article abstract in the fields of ophthalmology, neurology, and radiology. Among BERT, XGBoost, and logistic regression classifiers, the BERT model was the most accurate. Citation metrics may potentially be incorporated into future recommendation system algorithms. Further, artificial intelligence approaches to journal recommendation are evolving with the potential to increase the efficiency of the publication process for researchers.

## Abbreviations

BERT: Bidirectional Encoder Representations from Transformers
CBGJRS: Content and Bipartite Graph to Recommend Journals for Submission
MeSH: Medical Subject Headings
MTE: mixtures of transformer encoders
PSRMTE: paper submission recommendation using mixtures of transformer encoders
S2RSCS: Scientific Submission Recommendation System for Computer Science
TF-IDF: term frequency-inverse document frequency

## Data Availability

The data used in this study are freely accessible using the PubMed application programming interface.
